# Impact of Manufacturing Stages and Processing Scales on the Microbial Profile of Hurood

**DOI:** 10.3390/foods15132261

**Published:** 2026-06-24

**Authors:** Tong Chen, Yuan Niu, Yongchao Pan, Xiaoying Zhang, Lianyixin Liu, Shuhui Pang, Ying Zhao, Caiyun Wang, Nan Wu, Hong Zhu, Yue Cui

**Affiliations:** 1 School of Food Science and Biology, Hebei University of Science and Technology, Shijiazhuang 050018, China; chentong0429@163.com (T.C.); 18031381461@163.com (Y.N.); 15031752469@139.com (Y.P.); 13303173451@163.com (L.L.); pangshuhuishandong@163.com (S.P.); nndwunan@163.com (N.W.); 2 National Technology Innovation Center for Dairy, Hohhot 010110, China; zhangxiaoying6@yili.com (X.Z.); zhaoying8@yili.com (Y.Z.); wangcaiyun@yili.com (C.W.)

**Keywords:** hurood, manufacturing stages, processing scales, cheese microbiology, cheese quality

## Abstract

Traditional cheese products harbor complex microbial communities that influence their quality and safety. However, the effects of processing scale and manufacturing stage on the microbial profile of hurood, a traditional Mongolian cheese, remain poorly understood. This study examined microbial indicators, community composition, and succession dynamics across four manufacturing stages (raw milk, yogurt, whey, and hurood) and three processing scales (pastoral household, workshop, and factory) using plate counting and 16S rRNA gene amplicon sequencing. Twenty-four samples were collected from Xilin Gol, Inner Mongolia. Total aerobic plate counts and coliform counts decreased significantly from raw milk (7.30 and 4.49 log CFU/g, respectively) to hurood (2.02 and 0.34 log CFU/g, respectively; *p* < 0.05), reflecting progressive microbial reduction through acidification and thermal treatment, whereas yeast counts remained stable across stages. Firmicutes dominated the fermented stages, with *Lactococcus* and *Lactobacillus* as the predominant genera. Whey harbored an exceptionally high abundance of *Acetobacter* (21.6%), highlighting its valorization potential. Factory-scale production yielded the lowest mold and coliform counts in finished products despite higher initial coliform levels in industrial raw milk, reflecting the effectiveness of standardized hygiene management. In contrast, workshop-scale samples exhibited a higher relative abundance of environmental indicator bacteria, suggesting a comparatively elevated contamination risk this intermediate production scale. PICRUSt2-based functional predictions indicated stage-specific metabolic potential, including predicted enrichment of pyruvate and fatty acid metabolism in yogurt, amino acid metabolism in whey, and vitamin B6 metabolism in hurood. These findings provide a systematic microbial baseline for hurood, identify scale-specific microbiological risk profiles, and offer a foundation for targeted hygiene control and standardized production strategies.

## 1. Introduction

Traditional dairy products (e.g., cheese) produced in Inner Mongolia, China, have a long history [1]. Hurood, a traditional cheese prepared from raw milk using distinctive Mongolian artisanal techniques, is an indispensable component of the daily diet of local populations in Inner Mongolia and is deeply entrenched in the region’s ethnic food culture [2]. Owing to its unique flavor and high nutritional value, hurood is highly favored by local consumers [3]. Unlike most Western cheeses, hurood is produced through a unique simmering process using naturally acidified milk, without any addition of chymosin. This process relies on the combined effects of acidification and heating, which induce the denaturation and coagulation of casein [4]. Previous studies on hurood have primarily focused on its microbial diversity, its sensory characteristics, process optimization, and the probiotic potential of its *Lactobacillus* strains [5,6,7]. This interest in the microbial diversity of hurood stems from the coordinated and complex microbial interactions that drive its fermentation [8]. By metabolizing substrates such as lactose, other carbohydrates, fats, proteins, and peptides, these microorganisms generate short peptides, free amino acids, and a diverse array of aromatic compounds. Such biochemical processes not only facilitate the development of a unique flavor profile in hurood but also enhance the nutritional value of the final product [1,9].

Given that hurood is a naturally fermented product, its microbial communities are primarily derived from raw milk and the environment. Previous studies have demonstrated that these microbial communities play a critical role in food fermentation by directly influencing the quality, physicochemical properties, and unique characteristics of fermented products [10]. Several factors have been reported to affect the microbial composition of hurood and other traditional cheeses, including geographic region [11], season of production [12], and ripening time [13]. Previously, Guo et al. [2] documented the dynamic changes in microbial communities during hurood production and reported a decline in microbial diversity throughout the manufacturing process. However, they did not include the microbial diversity of whey, a byproduct that is subsequently utilized in beverages or powdered products [14,15].

Beyond the aforementioned factors, different processing conditions may also impact the quality and microbial composition of hurood, but so far, these effects have not been systematically characterized. Preliminary investigations have identified three major production scales for hurood manufacturing (Table 1): the pastoral household level, the small workshop level, and the industrial level [16]. In pastoral households, hurood production relies heavily on traditional practices with minimal hygiene control, leading to the introduction of undefined microorganisms from raw milk and the environment, the absence of strict processing parameters, and an increased risk of contamination and proliferation of harmful bacteria, all of which may compromise product quality and safety [7]. Small workshops offer improved hygiene conditions and greater product diversity through dedicated processing spaces and small-scale equipment. However, previous studies have shown that heat treatment of raw milk does not necessarily guarantee superior microbial quality, and batch-to-batch variations in flavor and sensory properties persist [17]. In contrast, industrial-scale production is characterized by standardized facilities, professional equipment, and quality inspection systems, enabling stricter hygiene management and GMP-compliant operations. Nevertheless, comparative studies evaluating microbial indicators and community composition across these three processing scales remain limited.

Therefore, the objective of this study was to examine microbial indicators, microbial community composition, and dynamic microbiota changes in hurood across different manufacturing stages (raw milk, yogurt, whey, and final hurood) and processing scales (pastoral household, workshop, and factory). This work aimed to fill critical knowledge gaps and provide a theoretical foundation for the standardized production and quality control of this traditional cheese.

## 2. Materials and Methods

### 2.1. Hurood Collection and Sampling

**Figure 1:**
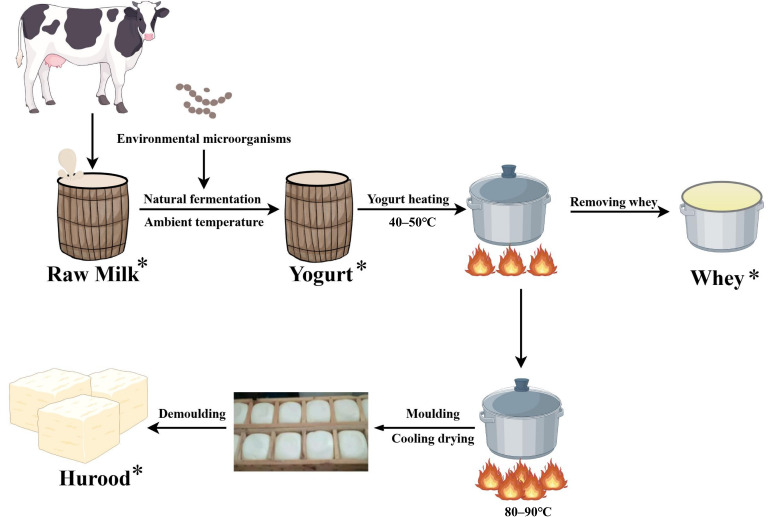
Flow diagram showing the steps of Hurood production. * indicates the four sampling stages used in this study.

Hurood samples were collected from the Xilin Gol region of Inner Mongolia. The traditional manufacturing process of hurood is illustrated in Figure 1. Raw milk was first naturally fermented at ambient temperature for 16–48 h, during which environmental microorganisms acidified the milk and induced coagulation. The resulting yogurt was then transferred to a pot and heated to 40–50 °C under continuous stirring. After complete separation of the whey and curd, the whey fraction was collected. The remaining curd was subsequently heated to 80 °C with constant stirring until it fully melted. This mixture was subsequently poured into wooden molds for shaping. Samples of the final hurood product were collected after natural cooling.

A total of 24 samples were obtained and categorized according to either the manufacturing stage or production scale. As shown in Figure 1, samples were collected during four manufacturing stages: raw milk (*n* = 6), yogurt (*n* = 6), whey (*n* = 6), and final hurood (*n* = 6). Alternatively, the samples were grouped according to production scale, as follows: pastoral household (*n* = 8), workshop (*n* = 8), and factory (*n* = 8). All samples were rapidly frozen at −20 °C, transported to the laboratory within 48 h under dry ice refrigeration, and subsequently stored at −80 °C until further analysis.

### 2.2. Physicochemical Measurement of Hurood Samples

The pH of each sample was measured at ambient temperature using a calibrated pH meter (model FE-28, Mettler Toledo, Greifensee, Switzerland), and water activity was determined using an HD-7 Water Activity Meter (Wuxi Huake Instrument & Meter Co., Ltd., Wuxi, China). All measurements were performed in triplicate, and the mean values were reported.

### 2.3. Microbial Analysis

Microbiological analyses, including total aerobic plate counts, yeast and mold counts, and coliform counts, were performed according to a previously described method with minor modifications [18]. Briefly, each sample (25 g) was transferred into a sterile sampling bag (Hunan DETE Biotechnology Co., Ltd., Changsha, China), and 75 mL of 0.1% peptone water was added. Samples were homogenized using a stomacher (Seward Stomacher Lab System 400, Worthing, England) at normal speed for 1 min. Appropriate serial dilutions of the homogenates were prepared, and aliquots (0.1 or 2 mL) were inoculated onto Petri dishes containing the appropriate culture media. Plate Count Agar (PCA; Beijing Land Bridge Tech Co., Ltd., Beijing, China) was used for enumerating the total aerobic bacteria, while Rose Bengal Chloramphenicol Agar (RBCA; Beijing Land Bridge Tech Co., Ltd., Beijing, China) was used for yeast and mold enumeration. PCA plates were incubated at 37 °C for 48 h, whereas RBCA plates were incubated at 28 °C for 5 days. For coliform enumeration, 2 mL aliquots of the appropriate dilutions were pour-plated using Violet Red Bile Agar (VRBA; Beijing Land Bridge Tech Co., Ltd., Beijing, China), and the plates were incubated at 30 °C for 18–24 h. Presumptive coliform colonies were subsequently transferred into Brilliant Green Lactose Bile Broth (BGLB; Beijing Land Bridge Tech Co., Ltd., Beijing, China) and incubated at 36 °C for 24 h. Gas production was considered indicative of a positive coliform reaction. After incubation, colonies were counted, and the results were expressed as log colony-forming units per gram of sample (log CFU/g). The detection limit of the plate count assay was 0.3 log CFU/g. All samples and plating procedures were performed in duplicate.

### 2.4. Analysis of Microbial Diversity in Hurood

#### 2.4.1. DNA Extraction and Amplicon Generation

Total genomic DNA was extracted from each sample using a commercial DNA isolation kit (TIANGEN Biotech, Beijing, China) according to the manufacturer’s protocol. DNA concentration and purity were assessed using a NanoDrop OneC spectrophotometer (Thermo Scientific, Waltham, MA, USA). The V3–V4 hypervariable region of the bacterial 16S rRNA gene was amplified using the primers 341F (5′-CCTACGGGNGGCWGCAG-3′) and 806R (5′-GGACTACNNGGGTATCTAAT-3′). PCR amplification was performed in 30 μL reaction mixtures containing 15 μL High-Fidelity PCR Master Mix (New England Biolabs, Ipswich, MA, USA), 0.2 μM each of forward and reverse primers, and approximately 10 ng of template DNA. The thermocycling conditions were as follows: initial denaturation at 98 °C for 1 min; 30 cycles of denaturation at 98 °C for 10 s, annealing at 50 °C for 30 s, and extension at 72 °C for 30 s; followed by a final elongation step at 72 °C for 5 min. PCR amplicons were pooled in equimolar concentrations and purified using a TIANgel Purification Kit (TIANGEN Biotech, Beijing, China). PCR amplicon integrity was verified using the Agilent 5400 Fragment Analyzer (Agilent Technologies, Santa Clara, CA, USA). Representative electropherograms of the 16S rRNA V3–V4 region and the ITS1 region from a representative sample are provided in Supplementary Figure S1, confirming successful amplification at the expected fragment sizes. Sequencing libraries were subsequently generated using the TIANSeq Fast DNA Library Prep Kit (Illumina) (TIANGEN Biotech, Beijing, China). Library quality was assessed using the Qubit@ 2.0 Fluorometer (Thermo Scientific, USA) and an Agilent 2100 Bioanalyzer system.

#### 2.4.2. High-Throughput Sequencing and Bioinformatic Analysis

Amplicon sequencing of the 16S rRNA gene libraries was performed on an Illumina NovaSeq X platform. Paired-end reads were processed using the DADA2 pipeline implemented in QIIME2 to infer amplicon sequence variants (ASVs) [19]. Taxonomic annotation of bacterial ASVs was conducted against the SILVA reference database (release 138) using a 99% similarity threshold. Bioinformatic analyses were performed in R version 4.1. ASV tables were managed and analyzed using the phyloseq package [20]. Alpha diversity indices and shared ASVs among samples were visualized using the VennDiagram package [21]. Beta diversity, assessed based on community structure, was analyzed using principal coordinate analysis (PCoA) via the vegan package [22]. The relative abundances of dominant taxa were visualized using the ggplot2 package in R (Version 3.6.2).

### 2.5. Statistical Analysis

Data were presented as mean ± standard deviation. To compare microbial counts across different manufacturing stages and processing scales, separate statistical analyses were performed for each factor due to the differences in data distribution. For manufacturing stages, data that did not meet the assumptions of normality or homoscedasticity (tested using Shapiro–Wilk and Levene’s tests) were analyzed using the nonparametric Kruskal–Wallis test. When overall differences were detected (*p* < 0.05), Dunn’s post hoc test with Bonferroni correction was applied for pairwise comparisons. For processing scales, data that satisfied the assumptions of normality and homoscedasticity were analyzed using one-way analysis of variance (ANOVA), followed by Tukey’s honestly significant difference (HSD) test for post hoc pairwise comparisons when the overall outcomes was significant (*p* < 0.05). The results of normality (Shapiro–Wilk) and homoscedasticity (Levene’s) tests for all microbial count parameters are summarized in Supplementary Table S1, along with the corresponding statistical tests applied. All statistical analyses were conducted using IBM SPSS Statistics version 26.0. Statistical significance was set at a two-tailed *p* < 0.05 for all analyses. Pairwise Spearman’s correlation analyses between microbial indicators and physicochemical parameters, as well as among microbial taxa, were performed using the psych package in R. Figures were generated using OriginPro 2018 (OriginLab Corporation, Northampton, MA, USA).

## 3. Results

### 3.1. Microbial Analysis of Traditional Hurood at Different Manufacturing Stages and Processing Scales

**Figure 2:**
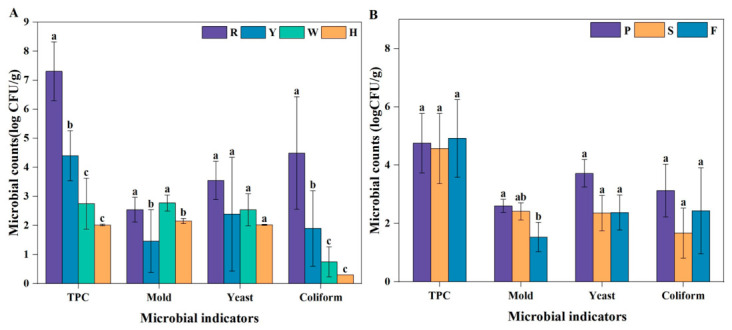
Microbiological analysis of traditional hurood at different manufacturing stages (**A**) and different processing scales (**B**). TPC: total aerobic plate counts. For manufacturing stages: R: raw milk; Y: yogurt; W: whey; H: Hurood. For processing scales: P: pastoral household; S: small workshop; F: factory. Different letters above the bars indicate significant differences within the same microbiological indicators across different manufacturing stages or processing scales (*p* < 0.05).

Microbial counts were compared across four major manufacturing stages: raw milk, yogurt, whey, and hurood (Figure 2A). A significant reduction in the total aerobic plate count (TPC) was observed across the manufacturing stages (*p* < 0.05). Raw milk exhibited the highest TPC (7.3 log CFU/g), but this value decreased significantly after fermentation (4.4 log CFU/g, *p* = 0.01). Meanwhile, the final hurood product (2.0 log CFU/g) and whey (2.7 log CFU/g) showed the lowest TPC (*p* < 0.001). Coliform counts followed a similar declining trend, decreasing from 4.5 log CFU/g in raw milk to 1.9 log CFU/g in yogurt (*p* = 0.004), 0.7 log CFU/g in whey (*p* < 0.001), and 0.3 log CFU/g in hurood (*p* < 0.001). Mold counts were significantly higher in raw milk and whey than in yogurt and hurood samples (*p* < 0.05), whereas yeast counts did not differ significantly among stages (*p* > 0.05).

When samples were grouped according to production scale, no significant differences in TPC, yeast counts, or coliform counts were observed among pastoral household, workshop, and factory samples (*p* > 0.05; Figure 2B). However, factory samples exhibited significantly lower mold counts (1.5 log CFU/g) than pastoral household samples (2.6 log CFU/g; *p* = 0.018).

Because the representation of product types varied substantially across production scales, an additional analysis was conducted to evaluate the effects of production scale within each specific product category (Table 2). For raw milk, coliform counts were significantly higher in factory samples (6.2 log CFU/g) than in workshop (4.2 log CFU/g) or pastoral household samples (3.8 log CFU/g; *p* < 0.05), whereas the TPC, mold count, and yeast count did not differ significantly according to the processing scale. For yogurt, processing scale did not significantly affect the TPC (*p* > 0.05). However, mold counts were significantly lower in factory-produced yogurt (0.3 log CFU/g) than in pastoral household (2.4 log CFU/g, *p* = 0.004) and workshop (1.7 log CFU/g, *p* = 0.023) yogurt samples (*p* < 0.05). Yeast and coliform counts showed a similar trend, with significantly higher levels in pastoral household samples than in workshop and factory samples (*p* < 0.05). However, no significant differences were observed between the latter two groups (*p* > 0.05). For whey, the TPC and yeast count were comparable across processing scales, whereas mold counts were significantly lower in factory samples than in pastoral household and workshop samples (*p* < 0.05). Coliform counts decreased significantly from pastoral household-produced whey to workshop-produced whey and were undetectable in factory-produced whey. A similar trend was observed in hurood samples across the three different processing scales, except that coliforms were detected only in pastoral household-produced hurood.

### 3.2. Physicochemical Measurement of Hurood Samples

Raw milk exhibited the highest pH (6.56, Table 2), followed by the hurood (6.56 vs. 5.55, *p* = 0.041), yogurt (6.56 vs. 4.36, *p* < 0.012) and whey (6.56 vs. 3.76, *p* < 0.001), whereas no significant differences were observed among different processing scales for any product type (*p* > 0.05). Regarding water activity (Aw), raw milk and whey showed similar values; however, raw milk was significantly higher than yogurt (0.98 vs. 0.93, *p* = 0.038), while hurood exhibited the lowest Aw. Spearman’s correlation analysis revealed that TPC was positively correlated with pH (*r* = 0.663, *p* = 0.001) and water activity (*r* = 0.720, *p* < 0.001, Figure S2), and coliform counts were also positively correlated with pH (*r* = 0.574, *p* = 0.008) and water activity (*r* = 0.585, *p* = 0.007).

### 3.3. Microbial Alpha and Beta Diversity in Hurood Across Different Manufacturing Stages and Processing Scales

#### 3.3.1. Alpha Diversity of Microbial Communities

**Figure 3:**
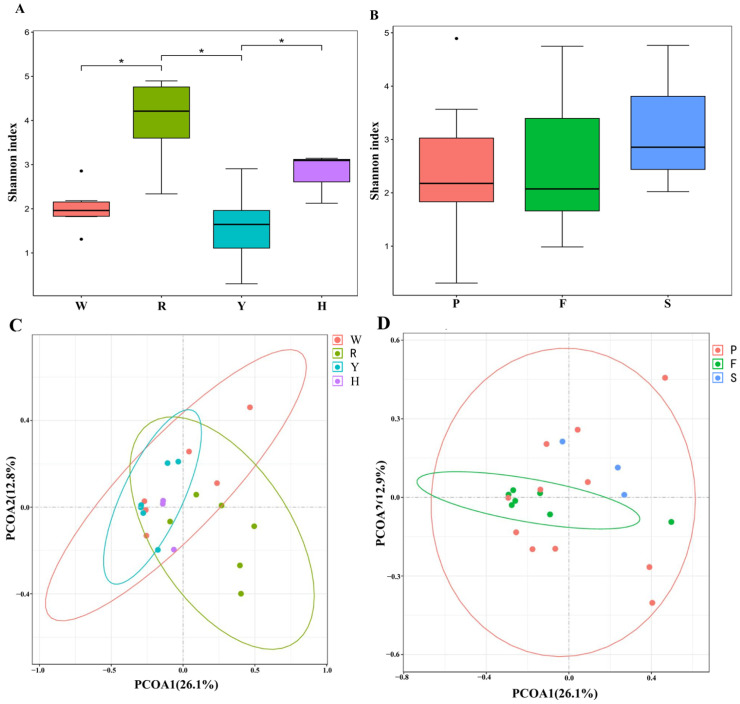
Alpha (**A**,**B**) and beta diversity (**C**,**D**) of hurood bacteria across different manufacturing stages (**A**,**C**) and different processing scales (**B**,**D**). R: raw milk, Y: yogurt, W: whey, H: hurood, P: pastoral household, S: workshop, F: factory. * indicates a significant difference among the groups (*p* < 0.05).

In this study, 16S rRNA gene sequencing was employed to investigate hurood microbial community dynamics across different manufacturing stages and processing scales. The Shannon alpha diversity index, which reflects both the richness and evenness of microbial communities, was used as the primary diversity metric. The results demonstrated a marked decrease in the Shannon index from raw milk to naturally fermented yogurt (*p* = 0.006) (Figure 3A). After the yogurt was heated and separated from whey during hurood production, the Shannon index of the final hurood product became significantly higher (*p* = 0.039) than that of the yogurt. Similarly, the alpha diversity of whey was significantly lower than that of raw milk (*p* = 0.006). When comparing different production scales, no significant differences in the Shannon index were observed among pastoral household (*p* = 0.72), workshop, and factory samples, indicating relatively similar microbial diversity across the three processing scales (Figure 3B).

#### 3.3.2. Beta Diversity of Microbial Communities

To evaluate differences and similarities among bacterial communities, beta diversity analysis was performed based on Bray–Curtis and weighted UniFrac distances. The results were visualized using PCoA. With regard to the manufacturing stages (Figure 3C), the first two principal coordinates explained 26.1% and 12.8% of the observed variation, respectively. The PCoA plot revealed a clear separation between raw milk samples and yogurt samples (*p* = 0.006), as well as between raw milk and hurood samples (*p* = 0.038). In contrast, the microbial communities of yogurt, whey, and hurood exhibited substantial overlap, indicating relatively similar community structures among these stages. In contrast, analysis of samples from different processing scales revealed no distinct clustering patterns (Figure 3D), suggesting that processing scale was not a major determinant of overall microbial community composition in the final product.

### 3.4. Abundance and Composition of Microbiota Across Hurood Manufacturing Stages and Processing Scales

#### 3.4.1. Phylum-Level Microbial Community Composition

**Figure 4:**
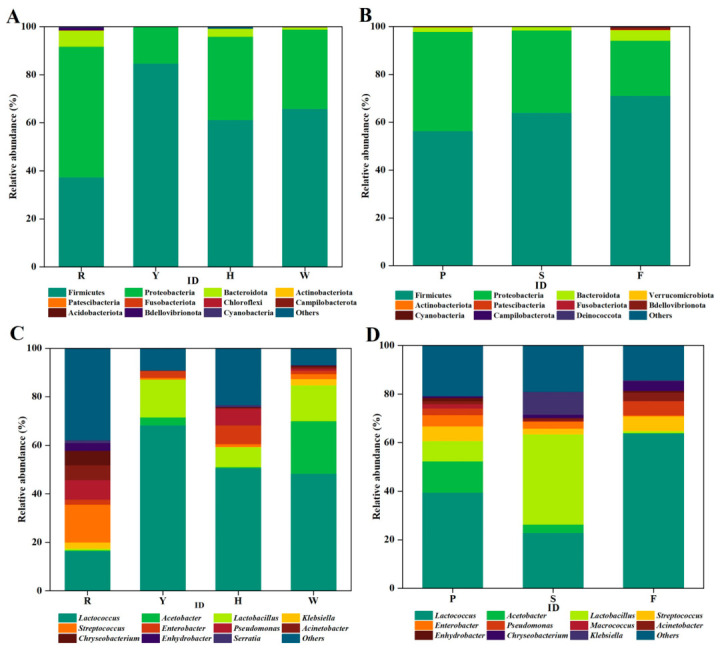
Relative abundance of microbes in hurood across different manufacturing stages at the phylum (**A**) and genus (**C**) levels. Relative abundance of microbes in hurood across different processing scales at the phylum (**B**) and genus (**D**) levels. R: raw milk, Y: yogurt, W: whey, H: hurood, P: pastoral household, S: workshop, F: factory.

Four bacterial phyla were detected across all samples obtained at different manufacturing stages: Firmicutes, Proteobacteria, Bacteroidota, and Actinobacteriota (Figure 4A). Raw milk was dominated by Proteobacteria (54.4%), followed by Firmicutes (37.4%) and Bacteroidota (6.7%). At the other three stages, Firmicutes showed a considerable predominance (>60%), reaching an abundance of 84.7% in yogurt. Bdellovibrionota (1.2%) and Deinococcus (0.4%) were unique to raw milk, whereas Fusobacteriota was exclusively detected in whey.

When considering the production scale, six phyla were identified across all samples: Firmicutes, Proteobacteria, Bacteroidota, Verrucomicrobiota, Actinobacteriota, and Patescibacteria (Figure 4B). Regardless of production scale, Firmicutes (56.5–71.2%) and Proteobacteria (23.1–41.4%) were consistently dominant across all samples.

#### 3.4.2. Genus-Level Microbial Community Composition

Eleven bacterial genera (*Lactococcus*, *Streptococcus*, *Acinetobacter*, *Chryseobacterium*, *Enhydrobacter*, *Enterobacter*, *Lactobacillus*, *Klebsiella*, *Pseudomonas*, *Serratia* and *Acetobacter*) were shared among samples from all manufacturing stages (Figure 4C). Raw milk harbored nine genera with a relative abundance >2%, including *Lactococcus* (16.4%), *Streptococcus* (15.5%), *Pseudomonas* (8.0%), *Acinetobacter* (6.2%), *Chryseobacterium* (5.9%), *Enhydrobacter* (3.1%), *Macrococcus* (3.1%), *Klebsiella* (2.6%), and *Enterobacter* (2.1%). Four such genera (>2%) were identified in yogurt: *Lactococcus* (68.3%), *Lactobacillus* (15.6%), *Acetobacter* (3.2%), and *Enterobacter* (2.8%). Meanwhile, *Lactococcus* (48.3%), *Acetobacter* (21.6%), *Lactobacillus* (14.9%), *Klebsiella* (2.5%), and *Streptococcus* (2.1%) were identified in whey. Additionally, *Lactococcus* (50.7%), *Lactobacillus* (8.2%), *Enterobacter* (7.8%), *Pseudomonas* (6.8%), and *Muribaculum* (2.6%) were identified in the final hurood product.

Eleven bacterial genera were identified in all samples across different production scales (Figure 4D). These were as follows: *Lactococcus*, *Acetobacter*, *Lactobacillus*, *Streptococcus*, *Enterobacter*, *Pseudomonas*, *Macrococcus*, *Acinetobacter*, *Enhydrobacter*, *Chryseobacterium*, and *Klebsiella*. Pastoral household samples were dominated by *Lactococcus* (39.5%) and *Acetobacter* (12.9%). In workshop samples, *Lactobacillus* (37.3%) was the most abundant, followed by *Lactococcus* (22.8%). Meanwhile, the dominant genera in factory samples were *Lactococcus* (64.0%) and *Streptococcus* (5.9%).

#### 3.4.3. Analysis of Microbial Differences Across Different Manufacturing Stages and Production Scales

**Figure 5:**
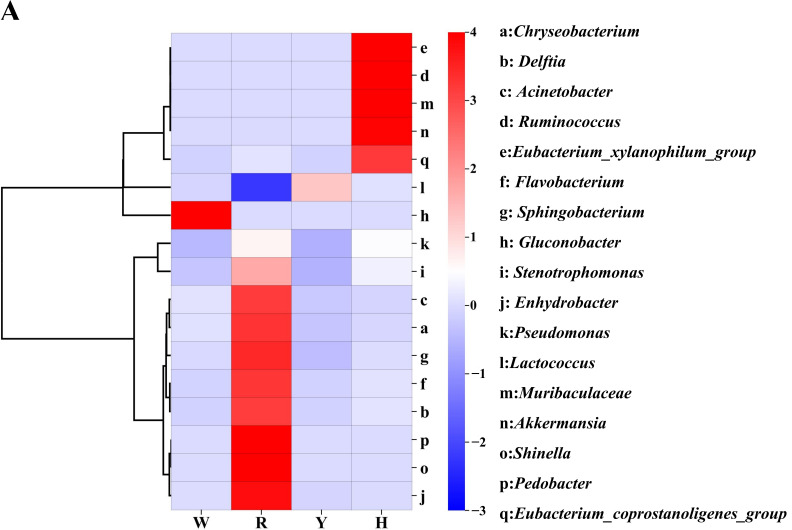

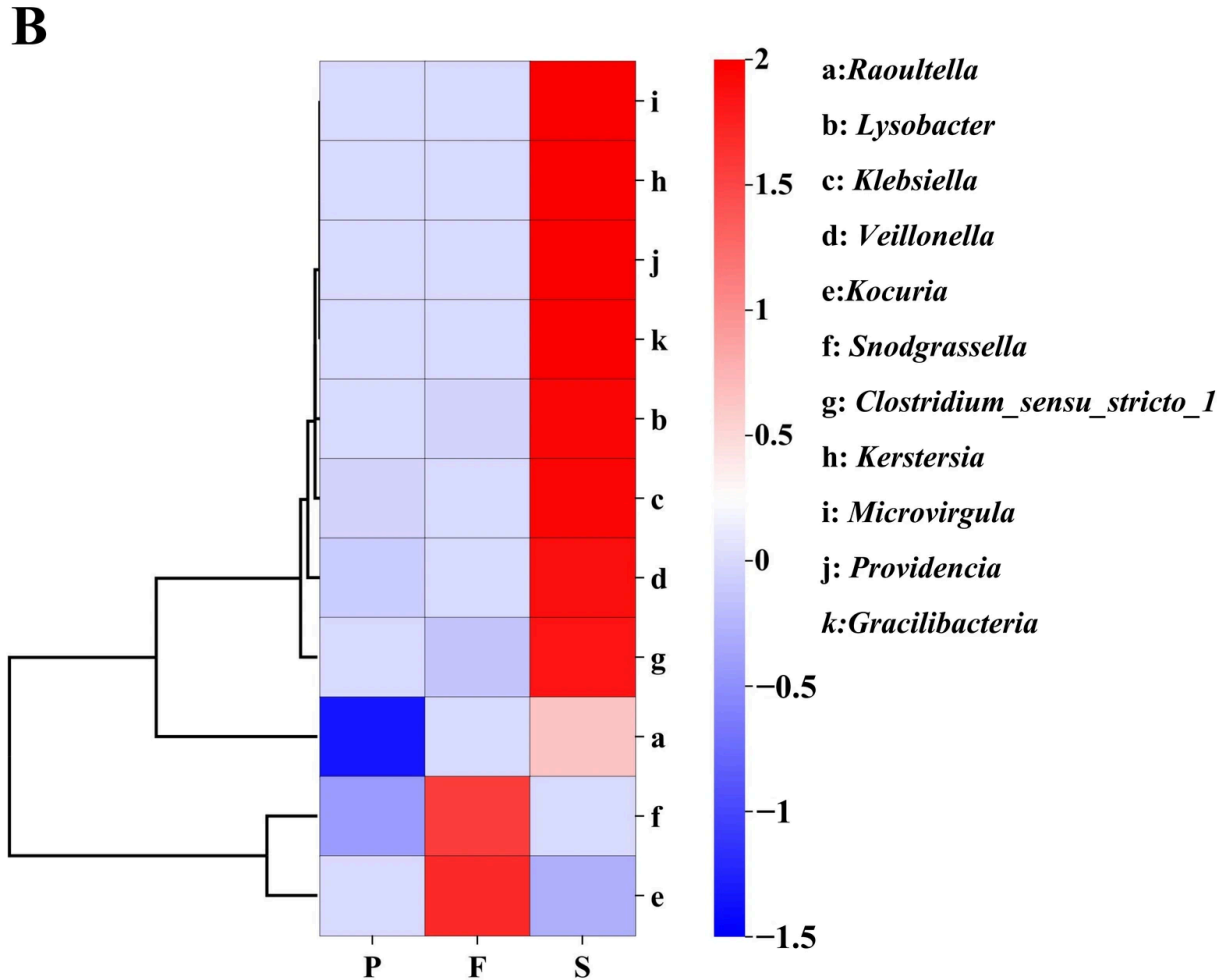
Cluster heatmap showing microbial differences across the four manufacturing stages (**A**) and three production scales (**B**). R: raw milk, Y: yogurt, W: whey, H: hurood; P: pastoral household, S: workshop, F: factory.

Genus-level differences were analyzed among the four manufacturing stages and three production scales. Across the manufacturing stages, nine genera—including *Enhydrobacter*, *Shinella*, *Pedobacter*, *Delftia*, *Flavobacterium*, *Sphingobacterium*, *Chryseobacterium*, *Acinetobacter*, and *Stenotrophomonas*—were enriched in raw milk (Figure 5A). Five genera, namely, *Eubacterium_coprostanoligenes_group*, *Akkermansia*, *Muribaculaceae*, *Ruminococcus*, and *Eubacterium_xylanophilum_group*, were enriched in the final hurood samples. Notably, *Lactococcus* abundance was significantly lower in raw milk than in the other fermented products. Additionally, *Gluconobacter* showed its highest abundance in whey.

With regard to processing scale, eight genera, including *Clostridium_sensu_stricto_1*, *Veillonella*, *Klebsiella*, *Lysobacter*, *Gracilibacteria*, *Providencia*, *Kerstesia*, and *Microvirgula*, showed significantly higher abundance levels in small workshop samples (Figure 5B). In contrast, factory-produced samples exhibited higher abundance levels of *Snodgrassella* and *Kocuria*. Meanwhile, the abundance of *Raoultella* was lower in pastoral household samples than in small workshop and factory samples.

#### 3.4.4. Correlation Analysis Among Dominant Strains at the Genus Level

**Figure 6:**
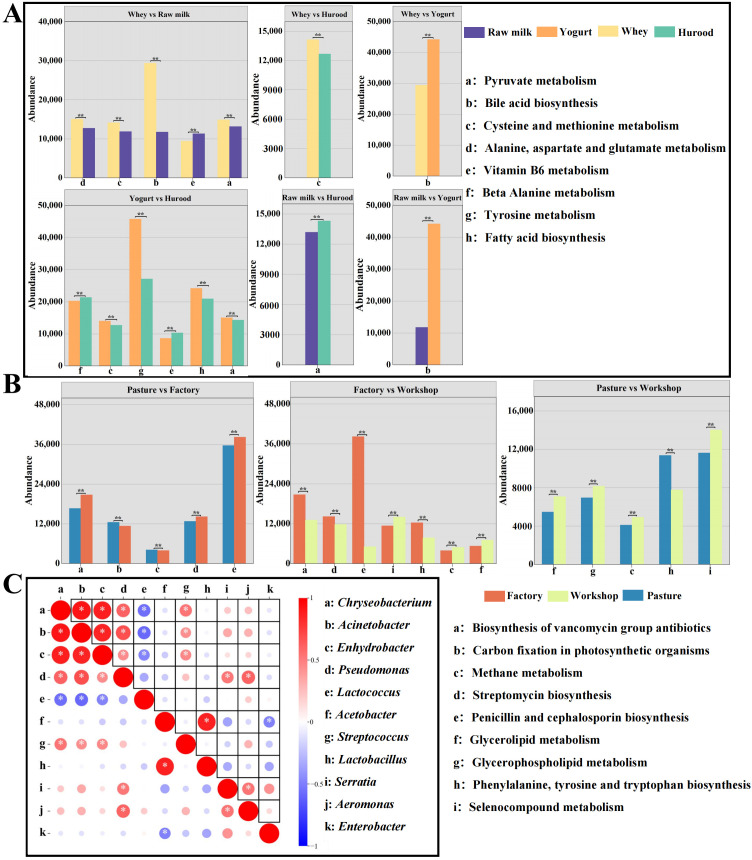
Differential analysis of metabolic pathways in hurood samples across different manufacturing stages (**A**) and production scales (**B**). Correlation analysis of dominant bacteria at the genus level (**C**). The size of the circle increases with the absolute correlation coefficient, and the color of the circle indicates the nature of the correlation. Significance is denoted as follows: * for *p* < 0.05, ** for *p* < 0.01.

The relationships among the dominant bacteria (present in all samples and with >0.2% abundance during at least two manufacturing stages) were analyzed through Spearman’s correlation analysis (Figure 6C). Significant positive correlations (*p* < 0.05) were observed among *Chryseobacterium*, *Acinetobacter,* and *Enhydrobacter*, all of which were negatively correlated with *Lactococcus* (*p* < 0.05). Meanwhile, *Pseudomonas* and *Streptococcus* were positively correlated with *Chryseobacterium*, *Acinetobacter*, and *Enhydrobacter* (*p* < 0.05). Additionally, *Pseudomonas* showed a positive correlation with *Serratia* and *Aeromonas*. Finally, *Acetobacter* was positively correlated with *Lactobacillus* but negatively correlated with *Enterobacter* (*p* < 0.05).

### 3.5. Predicted Metabolic Pathways Across Different Sample Types

PICRUSt2 (Phylogenetic Investigation of Communities by Reconstruction of Unobserved States) analysis predicted distinct functional potential profiles among the different hurood samples. Notably, the pyruvate metabolism pathway showed the highest predicted enrichment in yogurt, followed by whey and hurood, with the lowest enrichment observed in raw milk (Figure 6A). Similarly, the bile acid biosynthesis pathway was predicted to be significantly more enriched in yogurt than in the other three sample types (yogurt vs. raw milk: *p* < 0.001; yogurt vs. hurood: *p =* 0.02; yogurt vs. whey: *p =* 0.04). Regarding amino acid metabolism, the cysteine and methionine metabolism pathway, as well as the alanine, aspartate and glutamate metabolism pathway, were predicted to be significantly more enriched in whey than in raw milk (*p* = 0.007) and hurood (*p* = 0.009). In contrast, beta-alanine metabolism was predicted to be more enriched in hurood than in yogurt (*p* = 0.039). For lipid metabolism, the fatty acid biosynthesis pathway was significantly more enriched in yogurt than in hurood (*p* = 0.02). Additionally, vitamin B6 metabolism showed significantly higher predicted enrichment in hurood than in yogurt (*p* = 0.02) and in raw milk than in whey (*p* = 0.02).

Distinct predicted functional profiles were also observed across the three processing scales (Figure 6B). Factory samples were predicted to exhibit significantly greater enrichment of pathways related to the biosynthesis of vancomycin group antibiotics, streptomycin biosynthesis, and penicillin and cephalosporin biosynthesis when compared to samples from pastoral households and workshops (*p* < 0.05). Conversely, pathways associated with carbon fixation in photosynthetic organisms and methane metabolism showed lower enrichment in factory samples (*p* < 0.05). Workshop samples exhibited significant enrichment of glycerolipid, glycerophospholipid, and selenocompound metabolism, but showed the lowest enrichment of phenylalanine, tyrosine, and tryptophan biosynthesis (*p* < 0.05). However, it must be emphasized that all functional inferences in this section are PICRUSt2-based predictions, which are computationally inferred from 16S rRNA-derived taxonomic composition using reference genome databases. These predictions do not measure actual gene expression, enzyme activity, or metabolite production, and require validation through shotgun metagenomics, metatranscriptomics, or metabolomics.

## 4. Discussion

The microbial profile of traditional hurood is shaped by microorganisms originating from raw milk and the surrounding environment [12], as well as by processing parameters such as temperature and pH [23,24]. In this study, the microbiological profiles of raw milk, yogurt, whey, and hurood at different manufacturing stages were compared across the pastoral household, small workshop, and industrial level, providing a multi-scale and multi-stage microbial portrait of this traditional cheese. Raw milk exhibited significantly higher TPCs and coliform counts than the subsequent fermented stages, whereas whey and final hurood showed the lowest microbial loads (*p* < 0.05). The high initial microbial loads were consistent with the well-documented contamination of raw milk from animal skin, udders, and the milking environment [25]. The progressive decline was attributable to two sequential barriers operating along the production stage. First, the spontaneous fermentation of raw milk rapidly reduced the pH from 6.56 in raw milk to 4.36 in yogurt (*p* < 0.001; Table 2), a threshold at which the growth of most enteric bacteria, including coliforms, was substantially inhibited. Notably, the pH of whey was even lower (3.76. Table 2), which may reflect the accumulation of organic acid during fermentation and cooling. Second, the thermal treatment applied after whey separation further eliminated heat-sensitive vegetative cells, as also reported for traditional hurood by Guo et al. [2], who observed a progressive decline in microbial diversity and counts during the processing of traditional hurood.

Yeast and mold counts, however, followed different trends. Mold counts were significantly higher in raw milk and whey than in yogurt and hurood (*p* < 0.05), whereas yeast counts remained stable across all stages (*p* > 0.05). This discrepancy may reflect differences in the stress tolerance between these two fungal groups. Raw milk is highly susceptible to contamination by airborne mold spores [26], but the low pH and organic acids produced by lactic acid bacteria (LAB) during yogurt fermentation effectively inhibit the growth of molds [27]. Although both whey and hurood are exposed to air during processing, whey exhibited higher mold counts, which could be attributed to several factors: (i) whey separation at approximately 45 °C is insufficient to inactivate most mold spores, whereas hurood undergoes further heating to approximately 80 °C; (ii) consistent with the water activity measurements (Table 2), whey retained high water activity and abundant residual nutrients, providing favorable conditions for mold growth, while hurood exhibited the lowest aw among all stages, reflecting the effect of dehydration; and (iii) yeasts generally exhibit greater tolerance to low pH, moderate heat, and reduced water activity than molds, with many yeast species being capable of growing at pH 3.0–5.0 and aw 0.70–0.80 [28]. The stable yeast population observed throughout processing is noteworthy because yeasts play a consistent ecological role in flavor development and synergistic interactions with LAB [29,30].

A key finding of this study concerns the divergent microbial safety profiles across processing scales. Although raw milk from industrial plants exhibited significantly higher coliform counts than milk from pastoral households and workshops, the final industrial products showed substantially lower mold and coliform counts. The elevated coliform levels in industrial raw milk were likely the result of centralized milk collection from multiple farms with varying hygiene standards [18,31,32]. However, the three processing scales differ fundamentally in their production environment, equipment and hygiene management (Table 1). Factory-scale production operates in GMP-compliant workshops with temperature and humidity-controlled environments, automated equipment, and comprehensive quality management system, creating a multi-hurdle framework that effectively suppresses microbial proliferation. Importantly, pH and water activity did not differ significantly among the three processing scales for any product type (*p* > 0.05; Table 2), indicating that the scale-dependent differences in microbiological quality were attributable to hygiene management practices rather than to physicochemical conditions. In contrast, pastoral households and small workshops, despite the accumulated experience of traditional producers, often rely on open-vessel fermentation, manual handling, and ambient cooling, which introduce fewer and less reliable barriers [17]. Similar scale-dependent differences in microbiological quality have been reported in dairy and beef processing plants [33,34], supporting the findings that enhanced microbial safety is a robust advantage of standardized industrial-scale production.

In this study, 16S rRNA sequencing revealed a pronounced microbial succession dynamics during hurood manufacturing. Raw milk showed a significantly higher alpha diversity than yogurt, whey, and hurood, while hurood exhibited a higher diversity than yogurt (*p* < 0.05). This pattern likely reflects the rapid dominance of LAB during spontaneous fermentation, which lowered the pH and suppressed acid-sensitive microorganisms, reducing the alpha diversity. Subsequent processes such as reheating and whey removal likely enriched thermotolerant or thermophilic acid producing bacteria further [4], while open air cooling introduced environmental microorganisms, thus collectively increasing the microbial diversity of hurood. The discrepancy between our findings and those of Guo et al. [2], who reported no significant difference in diversity between hurood and yogurt, could be attributed to differences in environmental exposure, food-contact surfaces, or the duration of open-air drying [35,36].

At the genus level, a clear succession was observed. Raw milk showed either an abundance (>5%) or significant enrichment (*p* < 0.05) of potential spoilage bacteria such as *Pseudomonas*, *Acinetobacter*, and *Chryseobacterium*, as well as environmental bacteria (*Pedobacter* and *Delftia*). This profile was consistent with the well-documented contamination of raw milk by psychrotrophic bacteria from milking equipment, storage tanks, and farm environments [37,38]. Among these, *Pseudomonas* and *Acinetobacter* are of particular concern. As psychrotrophs, they can proliferate during refrigerated storage and secrete heat-stable extracellular lipases and proteases that survive pasteurization and subsequently degrade milk fat and casein [2,39], thereby causing off-flavors, textural defects, and reduced cheese yield [40,41]. Nevertheless, raw milk also contained *Lactococcus* (16.4%) and *Streptococcus* (15.5%), which serve as the natural inoculum driving subsequent acid-coagulation and fermentation.

Following fermentation, Firmicutes remained dominant, with *Lactococcus* and *Lactobacillus* being prevalent in yogurt, whey, and hurood, while *Acetobacter* was only highly abundant in whey, as reported for other fermented dairy products [2,11]. *Lactococcus* and *Lactobacillus* can not only inhibit the growth of spoilage bacteria but also contribute to flavor development and nutrient enrichment by producing short peptides, free amino acids, short-chain fatty acids, and aromatic compounds [42]. Notably, several genera, including *Akkermansia*, *Muribaculaceae*, and *Ruminococcus*, were significantly enriched in the final hurood relative to earlier stages. Although present at low relative abundance, their detection is biologically interesting. These microorganisms are commensal members of the rumen and hindgut microbiota and were likely introduced through direct contact during hand-milking or via cross-contamination from workers or the surrounding environment [13,43,44]. Their persistence through the fermentation may reflect not only physical carryover but also potential interactions. Recent evidence indicates that *Muribaculaceae* can engage in cross-feeding relationships with *Lactobacillus* in co-culture systems [45], suggesting that metabolic complementarity may contribute to their retention. Of particular interest, *Akkermansia* and *Ruminococcus* have been recognized to have probiotic potential, including the maintenance of intestinal barrier integrity and the production of short chain fatty acids [46,47]. Their presence in the final hurood product may suggest that traditional fermentation not only eliminates spoilage-associated risks but can also enrich potentially beneficial microorganisms. However, it must be emphasized that 16S rRNA gene amplicon sequencing detects DNA irrespective of cell viability, and the obligately anaerobic nature of these genera raises questions about their survival through aerobic processing steps. Their actual viability and functional contribution to hurood should therefore be verified using culture-dependent approaches under anaerobic conditions before any probiotic claims are advanced.

Whey, a byproduct of hurood production, exhibited a high relative abundance of *Acetobacter* (21.6%, Figure 4C) and a significant enrichment of *Gluconobacter* (*p* < 0.05, Figure 5A). The selective enrichment of these acetic acid bacteria in whey, rather than in yogurt or hurood, could be attributed to the unique physicochemical conditions of this byproduct. Whey presented the lowest pH among all manufacturing stages (pH 3.76, Table 2), combined with high water activity and oxygen availability during open-air drainage, creating an optimal niche for aerobic, acid-tolerant acetic acid bacteria. In contrast, although yogurt also exhibited a low pH (4.36), its closed fermentation vessel likely limited oxygen availability, thereby constraining Acetobacter proliferation; meanwhile, hurood’s moderate pH and markedly reduced water activity were less favorable for these aerobic taxa. Moreover, the residual lactose and lactic acid in whey further served as substrates for oxidative fermentation [48]. Indeed, whey can be used directly to produce whey vinegar, which has antimicrobial and antioxidant properties, through *Acetobacter* fermentation [15,49]. The high indigenous *Acetobacter* load in hurood whey suggests that its valorization could be achieved with minimal starter inoculation, reducing processing costs.

Across processing scales, *Lactococcus* predominated in pastoral household and factory samples, whereas *Lactobacillus* was dominant in workshop samples (Figure 4D). Although this study did not directly measure fermentation duration, this taxonomic divergence aligns with the well-established succession model in which *Lactococcus* rapidly proliferates during early stages of fermentation, and is gradually replaced by the more acid-tolerant *Lactobacillus* as lactic acid accumulates [50,51]. In pastoral households, processing is typically initiated soon after milk coagulation. Similarly, in factories, refrigerated tankers and controlled incubation systems maintain standardized fermentation durations. In contrast, workshops represent an intermediate processing scale, may experience extended fermentation owing to delays in milk collection, transportation under inadequate temperature control, or batch scheduling constraints, thereby favoring the enrichment of *Lactobacillus*. This interpretation is supported by the field investigation summarized in Table 1, which indicates that workshops lack the dedicated temperature-controlled fermentation systems available in factory settings. However, in the absence of direct fermentation time measurements in the present study, this interpretation must be treated as a hypothesis warranting targeted investigation. More critically, workshop samples harbored significantly higher abundances of environmental indicator bacteria, including *Clostridium*, *Veillonella*, *Klebsiella*, *Providencia*, and *Microvirgula*, than either pastoral household and factory samples (*p* < 0.05; Figure 5B). These genera have been consistently associated with fecal contamination, polluted water, soil, inadequately sanitized food-contact surfaces, and human handling in food processing environments [52,53]. Their selective enrichment in workshop samples, but not in pastoral household or factory samples, suggests that intermediate-scale production may exhibit the highest microbial risks. This pattern was further supported by the absence of the significant enrichment of these genera in factory samples, reflecting the effectiveness of industrial hygiene management and pathogen control. In addition, the vast and sparsely populated pastoral household environment, together with its associated natural environmental stresses, could act as a barrier against the proliferation of environmental contaminants [54].

Correlation analysis revealed a significant negative correlation between *Lactococcus* and environmental microorganisms, consistent with previous reports [2,55]. It is well established that *Lactococcus* achieves competitive inhibition via the rapid uptake of carbon sources, acid production, and bacteriocin release [56,57]. In contrast, the positive correlation between *Acetobacter* and *Lactobacillus* observed in the present study suggests the synergistic relationship of these two acid-tolerant bacterial taxa during fermentation. This conclusion is supported by previous studies demonstrating that these two genera can establish symbiotic fermentation through metabolic complementarity, such that the lactobionic acid by *Acetobacter* promotes *Lactobacillus* growth, and their synergistic interactions contribute to the production of flavor-related metabolites in fermented milk [58,59].

Comparison with previous studies on traditional fermented dairy products further highlights the distinctive design of the present work. Unlike most prior studies that focused exclusively on either manufacturing stages or processing scales in isolation, the present study simultaneously investigated both dimensions within a single traditional product and included whey as a distinct sampling stage. This multi-dimensional design allows the disentanglement of stage-specific and scale-specific microbial drivers, which had not been systematically addressed in the prior literature on hurood or closely related traditional cheeses. A comparative summary of the present study with selected previous works is provided in Supplementary Table S2.

PICRUSt2-based functional prediction revealed stage-specific enrichment patterns consistent with the metabolic capabilities of their respective dominant taxa. All functional inferences in this section represent predicted potential rather than demonstrated metabolic activity and require experimental validation. In yogurt, pyruvate metabolism, bile acid biosynthesis, and fatty acid biosynthesis pathways were significantly enriched. Pyruvate acts as a central node of carbohydrate metabolism in LAB [60] and can be converted into key flavor compounds such as diacetyl, acetaldehyde, and acetate [61]. Under conditions of acid stress, LAB redirect pyruvate metabolism from lactic acid production toward fatty acid biosynthesis [62]. The predicted enrichment of these pathways in yogurts is consistent with the known biochemical roles of the dominant LAB, suggesting elevated metabolic potential for flavor precursor generation at this stage. The enrichment of bile acid biosynthesis at this stage likely reflects the high bile acid hydrolase activities in the dominant LAB [63,64]. Furthermore, secondary bile acids can regulate energy production, glucose and lipid metabolism, inflammation, and host–microbiota interactions, promoting the production of beneficial metabolites [65]. Whey exhibited enrichment of cysteine–methionine metabolism and alanine–aspartate–glutamate metabolism, consistent with active proteolysis by LAB [66] releasing amino acids into this aqueous fraction. During the hurood stage, most pathways showed limited enrichment due to low water activity. The predicted potential for vitamin B6 metabolism was retained in hurood, consistent with the abundance of B vitamins in cheese-like products [67]. LAB isolated from traditional Iranian yogurt have demonstrated vitamin B6 production capacity [68].

Across production scales, factory samples showed predicted enrichment of several antibiotic biosynthesis pathways, including vancomycin-group antibiotics, streptomycin, and penicillin/cephalosporin, indicating a higher relative abundance of microbial taxa with the potential for antibiotic synthesis. On one hand, this enrichment could enhance stress resistance and product stability within the fermentation system, while the resulting metabolites could serve as potential sources of natural food preservatives. On the other hand, it may also raise concerns regarding the transfer of antibiotic resistance genes within dense industrial microbial communities [69,70].

## 5. Limitations of the Study

Several limitations of this study should be acknowledged. First, the sample size (n = 24) was relatively small, and all samples were collected from a single region (Xilin Gol), which may not fully represent the diversity of hurood production practices across Inner Mongolia. Second, the fermentation time and temperature for each production batch were not systematically recorded, limiting our ability to mechanistically link processing parameters to the observed differences in microbial community composition across scales. Third, the functional predictions generated by PICRUSt2 are based solely on 16S rRNA-inferred taxonomic composition and thus reflect potential functional capacity rather than actual gene expression or metabolite production. These predictions require further validation through integrated metatranscriptomic and metabolomic approaches, particularly for the antibiotic biosynthesis pathways enriched in factory samples. Finally, the detection of potentially beneficial anaerobes, such as *Akkermansia* and *Ruminococcus*, awaits culture-dependent confirmation of viability and functional characterization.

## 6. Conclusions

This study systematically characterized the microbial profiles of traditional hurood across four manufacturing stages and three production scales. Microbial succession during manufacturing was marked by a sharp reduction in total aerobic plate counts and coliform counts from raw milk (7.3 and 4.5 log CFU/g, respectively) to hurood (2.0 and 0.3 log CFU/g, respectively), whereas yeast counts remained relatively stable. Alpha diversity declined during the fermentation step but partially recovered in hurood, reflecting the microbial selection during fermentation and subsequent microbial reintroduction during heating and open-air cooling. Firmicutes dominated the fermented samples, with *Lactococcus* and *Lactobacillus* identified as the predominant genera. Whey exhibited an exceptionally high abundance of *Acetobacter* (21.6%), highlighting its potential as a fermentation byproduct for valorization. Regarding processing scale, factory production achieved superior microbiological safety profile in the final product, despite the higher coliform levels observed in industrial raw milk, reflecting the effectiveness of standardized hygiene management and pasteurization systems. In contrast, workshop-scale production without rigorous hygienic control showed the greatest enrichment of environmental indicator bacteria and thus poses the highest microbiological risk. These findings identify critical control points and scale-specific risks in hurood production and provide a foundation for scale-appropriate hygiene interventions that preserve product uniqueness while improving safety.

Future research should integrate multi-omics approaches across larger, multi-regional sample sets and include controlled fermentation experiments with time-series sampling to disentangle the relative contributions of processing parameters, environmental microbiota, and raw milk microbiomes to the final product community. Nevertheless, this study provides a comprehensive microbial baseline for traditional hurood, identifies critical control points and scale-specific microbiological risks, and establishes a foundation for standardized production strategies that preserve product uniqueness while improving safety and consistency. These findings can be applied to the development of scale-specific hygiene control plans, the tailoring of starter cultures to suppress spoilage or pathogenic microbes without altering traditional flavor, and the provision of evidence-based benchmarks for food safety authorities to certify artisanal hurood production.

## Figures and Tables

**Table 1:** Comparison of key attributes across three production scales for hurood.

Parameter ^a^	Pastoral Household	Workshop	Factory
Raw Milk Origin	Own pasture, stored at ambient temperature	From nearby pasture; transported without cold chain	From cooperative pasture; inspection by automatic analyzer upon arrival
Daily Processing Capacity (kg/d)	Variable mainly 5–10	100–150	1000–2000
Primary use	Self-consumption	Self-consumption, local sale	Fully packaged commercial distribution
Hygiene Control	None Depends on water, files, and operator experience	Moderate Dedicated cheese-making room with zoning	High GMP-compliant workshop with controlled temperature & humidity
Estimated relative cost	Almost None	Moderate basic facility + small equipment	High standardized factory + full QA infrastructure

^a^ Data were collected through field observations and interviews at each site.

**Table 2:** Microbial counts in raw milk, yogurt, whey, and hurood according to processing scales ^1^.

Sample Type	Processing Scale	Microbial Counts (log CFU/g)				Physicochemical	
		TPC ^2^	Molds	Yeasts	Coliforms	pH	Aw ^4^
Raw milk	Pastoral household	6.85 ± 1.41 ^a^	2.70 ± 0.00 ^a^	3.27 ± 0.42 ^a^	3.78 ± 1.48 ^b^	6.57 ± 0.00 ^A^	0.98 ± 0.00 ^A^
	Workshop	7.17 ± 1.20 ^a^	2.67 ± 0.35 ^a^	3.59 ± 0.86 ^a^	4.15 ± 1.04 ^b^	6.57 ± 0.03 ^A^	0.98 ± 0.00 ^A^
	Factory	7.95 ± 0.06 ^a^	2.20 ± 0.71 ^a^	3.78 ± 0.83 ^a^	6.21 ± 0.72 ^a^	6.54 ± 0.02 ^A^	0.99 ± 0.01 ^A^
Yogurt	Pastoral household	4.58 ± 1.10 ^a^	2.40 ± 0.85 ^a^	4.89 ± 0.16 ^a^	3.40 ± 1.05 ^a^	4.33 ± 0.04 ^C^	0.93 ± 0.00 ^B^
	Workshop	3.85 ± 1.20 ^a^	1.69 ± 0.69 ^a^	0.95 ± 0.07 ^b^	1.50 ± 0.00 ^b^	4.33 ± 0.05 ^C^	0.94 ± 0.00 ^B^
	Factory	4.77 ± 0.33 ^a^	0.30 ± 0.00 ^b^	1.34 ± 0.47 ^b^	0.80 ± 0.28 ^b^	4.38 ± 0.01 ^C^	0.93 ± 0.01 ^B^
Whey	Pastoral household	2.93 ± 1.18 ^a^	3.01 ± 0.40 ^a^	2.60 ± 0.00 ^a^	1.20 ± 0.06 ^a^	3.74 ± 0.05 ^D^	0.94 ± 0.00 ^AB^
	Workshop	2.77 ± 0.94 ^a^	2.94 ± 0.23 ^a^	2.54 ± 0.25 ^a^	0.30 ± 0.00 ^b^	3.78 ± 0.01 ^D^	0.96 ± 0.02 ^AB^
	Factory	2.52 ± 0.04 ^a^	2.30 ± 0.00 ^b^	2.47 ± 0.01 ^a^	- ^3^	3.75 ± 0.01 ^D^	0.96 ± 0.02 ^AB^
Hurood	Pastoral household	2.33 ± 1.18 ^a^	2.72 ± 0.40 ^a^	2.50 ± 0.20 ^a^	1.20 ± 0.06 ^a^	5.54 ± 0.03 ^B^	0.88 ± 0.04 ^C^
	Workshop	1.93 ± 0.00 ^a^	2.10 ± 0.00 ^a^	1.80 ± 0.16 ^a^	-	5.55 ± 0.04 ^B^	0.88 ± 0.02 ^C^
	Factory	1.83 ± 0.04 ^a^	1.60 ± 0.00 ^b^	1.71 ± 0.01 ^a^	-	5.57 ± 0.01 ^B^	0.89 ± 0.01 ^C^

^1^ Different lowercase letters indicate significant differences in the microbial counts within the same sample type across the different processing scales (*p* < 0.05). Different uppercase letters indicate significant difference in the physicochemical parameters across all processing stages and scales combined (global comparison). ^2^ TPC: total aerobic plate count. ^3^ -: below the detection limit (<0.3 log CFU/g). ^4^: Aw: water activity.

## Data Availability

The raw data supporting the conclusions of this article will be made available by the authors on request.

## Supplementary Materials

The following supporting information can be downloaded at: https://www.mdpi.com/article/10.3390/foods15132261/s1, Figure S1: Representative Agilent 5400 Fragment Analyzer electropherograms of PCR amplicons. (A) Bacterial 16S rRNA gene V3–V4 region; (B) fungal ITS1 region; Figure S2: Spearman’s correlation heatmap showing correlation between physicochemical parameters (pH, Aw) and microbial indicators (TPC, molds, yeasts, and coliforms) in hurood; Table S1: Normality and homoscedasticity test results for microbial count parameters across manufacturing stages and processing scales; Table S2: Comparative summary of the present study with selected previous works on microbial diversity in traditional fermented dairy products.

## Abbreviations

The following abbreviations are used in this manuscript:

TPC	total aerobic plate count
LAB	lactic acid bacteria
CFU	Colony-Forming Unit
Aw	water activity
