# Supplementary material for: Impact of Manufacturing Stages and Processing Scales on the Microbial Profile of Hurood
Source: Foods. 2026 Jun 24;15(13):2261. doi: 10.3390/foods15132261 (PMC13362000; doi:10.3390/foods15132261)
Supplement: Supplementary file 1 [file foods-15-02261-s001.zip › Supplementary Figure.pdf]

A.

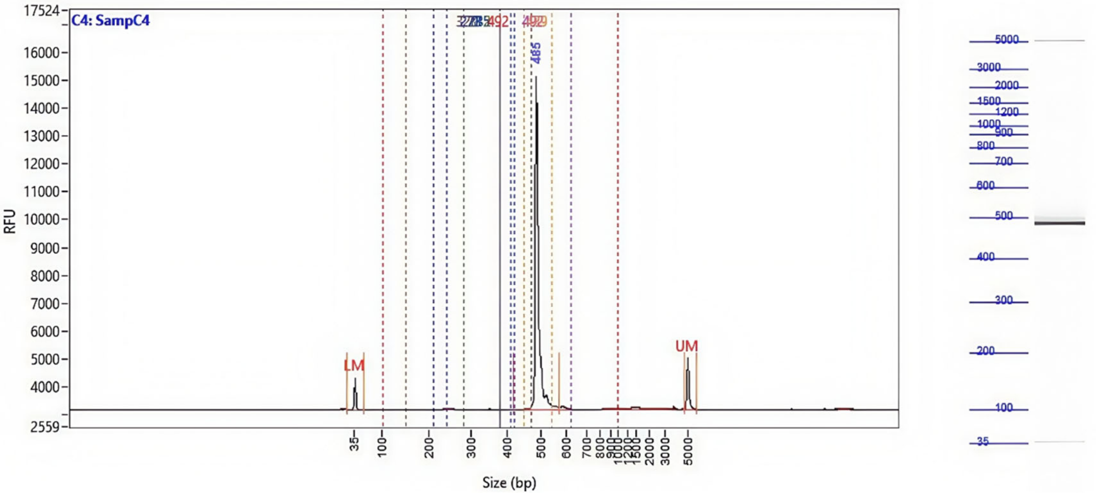

B.

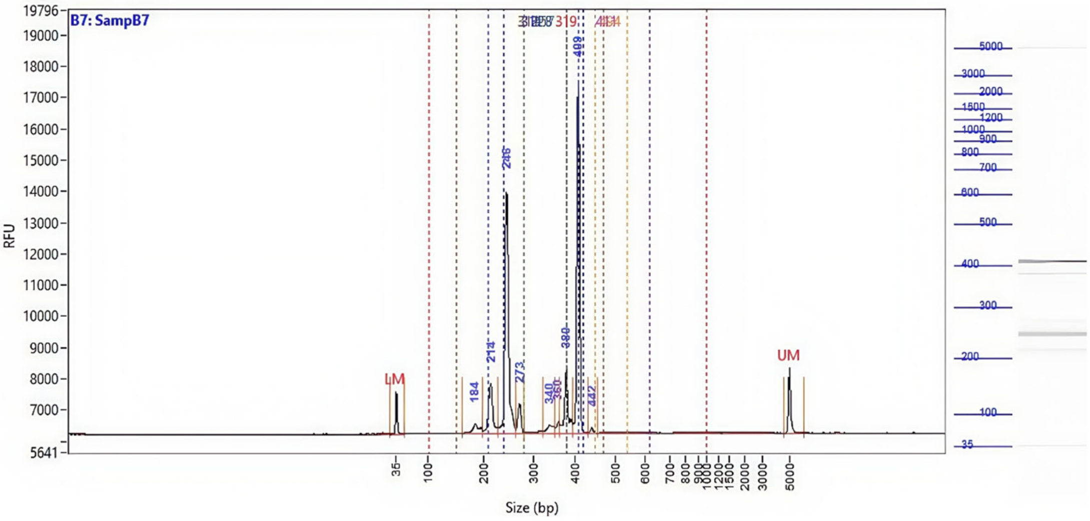

Supplementary Figure S1. Representative Agilent 5400 Fragment Analyzer electropherograms of PCR amplicons. (A) Bacterial 16S rRNA gene V3–V4 region; (B) fungal ITS1 region. x-axis: fragment size (bp); y-axis: relative fluorescence intensity (RFU).

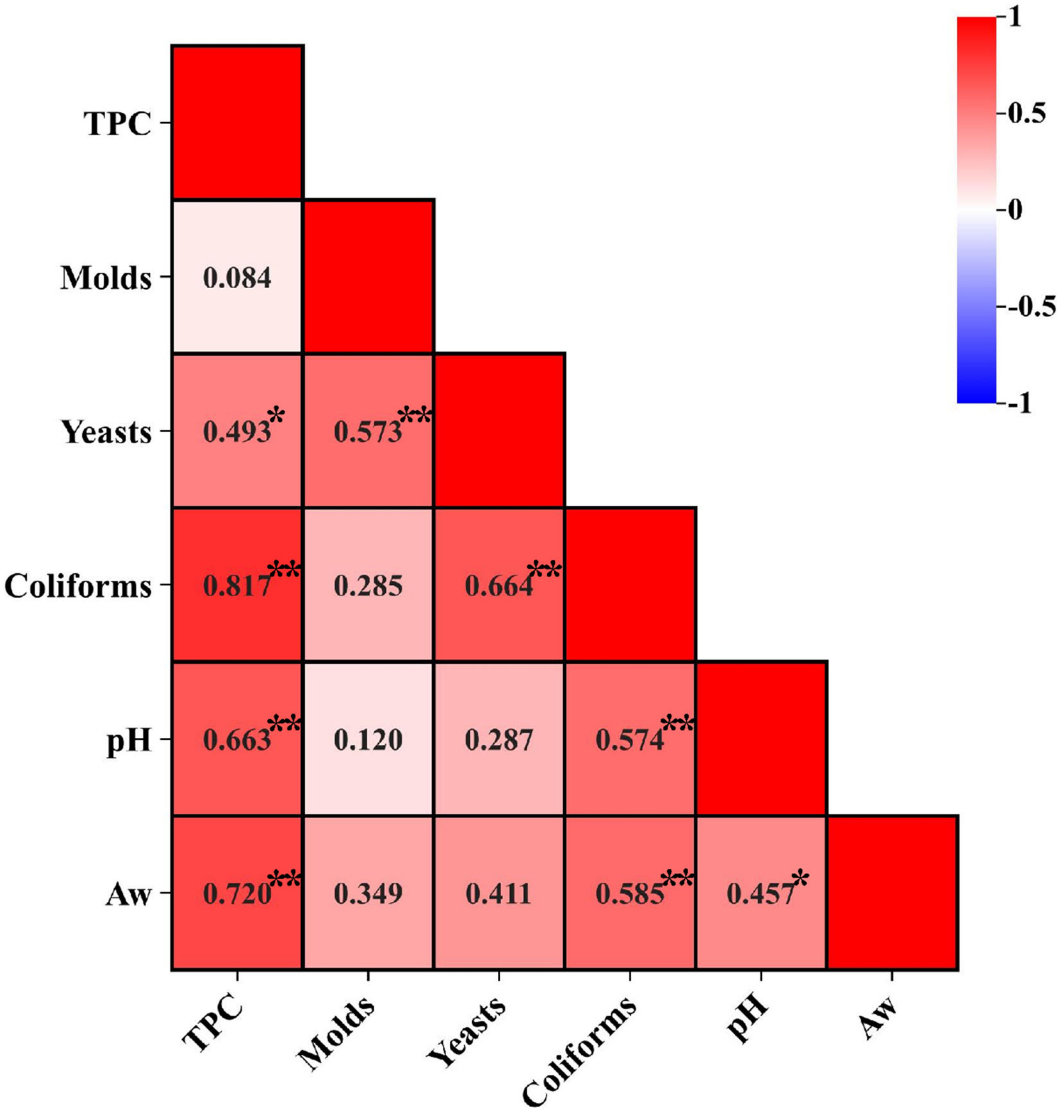

Supplementary Figure S2. Spearman's correlation heatmap showing correlation between physicochemical parameters (pH, Aw) and microbial indicators (TPC, molds, yeasts, and coliforms) in hurood. Color intensity indicates the strength of the Spearman correlation. Red indicates positive correlation, and blue indicates negative correlation. Numerical values represent correlation coefficients (*r*). Significance levels are indicated as $P < 0.05$ (*) and $P < 0.01$ (**) for two-tailed tests. Aw: water activity; TPC: total plate counts
