# Supplementary material for: Impact of Manufacturing Stages and Processing Scales on the Microbial Profile of Hurood
Source: Foods. 2026 Jun 24;15(13):2261. doi: 10.3390/foods15132261 (PMC13362000; doi:10.3390/foods15132261)
Supplement: Supplementary file 1 [file foods-15-02261-s001.zip › Supplementary Table.pdf]

Table S1. Normality and homoscedasticity test results for microbial count parameters across manufacturing stages and processing scales.[1]

Grouping factor	Parameter	*P* value of Shapiro-Wilk (normality)	*P* value of Levene's test (homoscedasticity)	Assumptions met?	Statistical test applied
Manufacturing stages	TPC[2]	0.124	0.04	No	nonparametric Kruskal–Wallis test, followed by Dunn’s post hoc test with Bonferroni correction for pairwise comparisons.
	Mold	0.046	0.009	No	
	Yeast	0.015	<0.001	No	
	Coliform	<0.001	<0.001	No	
Processing scales	TPC	0.24	0.776	Yes	one-way analysis of variance (ANOVA), followed by Tukey’s honestly significant difference (HSD) test for post hoc pairwise comparisons
	Mold	0.157	0.072	Yes	
	Yeast	0.546	0.885	Yes	
	Coliform	0.631	0.082	Yes	

[1] Normality was considered satisfied when Shapiro-Wilk $P \geq 0.05$; homoscedasticity was considered satisfied when Levene's $P \geq 0.05$. All statistical analyses were conducted using IBM SPSS Statistics version 26.0.

[2]TPC = total plate count (aerobic).

Table S2. Comparative summary of the present study with selected previous works on microbial diversity in traditional fermented dairy products.

Reference	Product	Region	Method	Key Findings	Difference from This Study
Guo et al.(2023)[1]	Hurood	Inner Mongolia, China	Metagenomic Sequencing	Declining microbial diversity during manufacturing; *Lactococcus* predominant.	Whey not included as a sampling stage; Single production scale.
Zhang et al.(2022)[2]	Mongolian cheese	Inner Mongolia, China	16S rRNA amplicon sequencing + Metabolomics	Bacterial succession correlated with flavor metabolite dynamics; *Lactococ cus* and *Lactobacill us* dominant throughout manufacturing	Metabolomic data included; no multi-scale processing comparison; whey not sampled
Zhao et al.(2021)[3]	Traditional Chinese cheeses	Multiple regions, China	16S rRNA amplicon sequencing	Manufacture process and geography shape microbial profiles.	Multiple cheese types pooled; no focus on hurood specifically; no processing scale comparison
Liang et al.(2021)[4]	Fermented dairy products	China, south Africa, Sri Lanka	16S rRNA amplicon sequencing	Regional differences in bacterial Community composition.	Cross-national comparison; no stage-specific or scale-specific analysis within a single product
Sun & D'Amico (2021)[5]	Farmstead cheese	USA	16S rRNA amplicon sequencing	Microbial succession driven by manufacturing environment and raw milk; enteric pathogens detected in some samples	Western-style cheese; no processing scale comparison
Present study	Hurood	Inner Mongolia, China	16S rRNA + ITS amplicon sequencing; PICRUSt2 functional prediction	Microbial succession across 4 stages; scale-specific safety profiles; whey harbors distinct microbiota with valorization potential	Simultaneous investigation of manufacturing stages × processing scales; whey included as distinct stage; first multi-scale comparison for hurood

Reference

1. Guo, S.; Da, L.; Li, L.; Li, B.; Wang, D.; Liu, W.; Menghe, B.; Chen, Y. Composition and changes of microflora in the manufacturing process of traditional hurood. *Lwt* **2023**, *181*, doi:10.1016/j.lwt.2023.114732.
2. Zhang, X.; Zheng, Y.; Feng, J.; Zhou, R.; Ma, M. Integrated metabolomics and high-throughput sequencing to explore the dynamic correlations between flavor related metabolites and bacterial succession in the process of Mongolian cheese production. *Food Res Int* **2022**, *160*, doi:10.1016/j.foodres.2022.111672.
3. Zhao, Z.; Ning, C.; Chen, L.; Zhao, Y.; Yang, G.; Wang, C.; Chen, N.; Zhang, Z.; Li, S. Impacts of manufacture processes and geographical regions on the microbial profile of traditional Chinese cheeses.

Food Res Int **2021**, *148*, doi:10.1016/j.foodres.2021.110600.

4. Liang, T.; Xie, X.; Zhang, J.; Ding, Y.; Wu, Q. Bacterial community and composition of different traditional fermented dairy products in China, South Africa, and Sri Lanka by high-throughput sequencing of 16S rRNA genes. *Lwt* **2021**, *144*, doi:10.1016/j.lwt.2021.111209.
5. Sun, L.; D'Amicoa, D.J. Composition, succession, and source tracking of microbial communities throughout the traditional production of a farmstead cheese. *Msystems* **2021**, *6*, 10.1128/msystems.00830-00821, doi:ARTN e00830-2110.1128/mSystems.00830-21.
